# An elderly patient with 17α-hydroxylase deficiency misdiagnosed as primary aldosteronism: a case report

**DOI:** 10.1186/s12902-022-01216-y

**Published:** 2022-12-02

**Authors:** Yuki Ishinoda, Asuka Uto, Yoshifumi Yamada, Maki Okazaki, Hidetomo Asada, Seina Wakamatsu, Isao Kurihara, Hironori Shibata, Tomohiro Ishii, Tomonobu Hasegawa, Hiroo Kumagai, Akira Kasuga

**Affiliations:** 1 Department of Endocrinology, National Defense Medical College, 3-2 Namiki, 359-8513 Tokorozawa-shi, Saitama, Japan; 2 Department of Pediatrics, Keio University School of Medicine, Tokyo, Japan

**Keywords:** 17-alpha-hydroxylase deficiency, 46XY testicular disorders of sex development, Adrenal insufficiency, Aldosterone, Case report, Congenital adrenal hyperplasia, Cortisol, Hypertension, Hypokalemia

## Abstract

**Background:**

17α-hydroxylase deficiency (17OHD) is a rare autosomal recessive disorder. Aldosterone levels are usually low in patients with 17OHD. However, among the approximately 150 cases of 17OHD reported to date, aldosterone levels were not low in all cases. Therefore, some 17OHD cases may have been misdiagnosed as primary aldosteronism (PA) cases. Often before puberty, 17OHD is diagnosed because of abnormal genital morphology and menstrual irregularities. However, we report a very rare case of 17OHD in an elderly patient with a high aldosterone/renin ratio (ARR) similar to that in PA.

**Case presentation:**

A 63-year-old Japanese woman was transferred to our medical facility for the evaluation of bilateral adrenal hypertrophy, which was incidentally discovered during an abdominal examination after cholecystectomy. The patient had hypokalemia and a high aldosterone/renin ratio. Her medical history included hypertension and right intracerebral capsular hemorrhage at the age of 30 years. Additional testing revealed low cortisol, high adrenocorticotropic hormone, and low testosterone and dehydroepiandrosterone sulfate, indicating congenital adrenal hyperplasia. Genetic analysis revealed a mutation in the *CYP17A1* gene and a karyotype of 46, XY; hence, she was diagnosed with 17OHD.

**Conclusion:**

17OHD can resemble PA. The combination of a high ARR and low cortisol level should trigger the consideration of 17OHD.

## Background

Congenital adrenal hyperplasia (CAH) is an autosomal recessive disorder caused by specific enzyme defects in the adrenal steroidogenic pathway [1]. The enzymes involved in steroidogenesis are mainly 21-hydroxylase, 11β-hydroxylase, and 17α-hydroxylase. The deficiency of 21-hydroxylase is the most frequent cause of CAH, accounting for approximately 95% of CAH, and it is caused by mutations in the gene encoding cytochrome P450 [2]. Mutations in the cytochrome P450 family 17 subfamily A member 1 (*CYP17A1*) gene result in a very rare form of CAH that causes 17α-hydroxylase/17,20-lyase deficiency (17OHD) [3]. The synthesis of androgens and cortisol from cholesterol involves 17α-hydroxylase/17,20 lyases; thus, the deficiency of these enzymes causes androgen and cortisol deficiency, respectively, and a mineralocorticoid precursor (11-deoxycorticosterone and corticosterone) excess. Low aldosterone levels in patients with 17OHD are thought to be due to 11-deoxycorticosterone and corticosterone because the mineralocorticoid effects of both steroids suppress the activity of the renin-angiotensin-aldosterone system [4]. However, among the approximately 150 cases of 17OHD reported to date, there were often cases wherein aldosterone levels were not low [5, 6]. Therefore, some cases of 17OHD may have been misdiagnosed as primary aldosteronism (PA). In addition, 17OHD is often diagnosed before puberty due to abnormal genital morphology and menstrual irregularities [6]. However, in the present case, we report a very rare case of 17OHD in an elderly patient with a high aldosterone/renin ratio (ARR) similar to that in PA. We also discuss the characteristics of genetic variants in previously reported cases of hyperaldosteronism and the impact of cross-reactivity on aldosterone levels.

## Case presentation

A 63-year-old woman was transferred to our medical facility for the evaluation of bilateral adrenal enlargement that was incidentally discovered during an abdominal examination after cholecystectomy. The patient was hypertensive and was taking an angiotensin II receptor blocker (candesartan) and a calcium channel blocker (nifedipine); she had a blood pressure of 142/82 mmHg. The referral source tests showed hypokalemia, and the ARR was very high. Thus, we initially considered a diagnosis of PA.

However, further investigation revealed that the patient had been diagnosed with hypertension at the age of 30 years and with left hemiplegia, alongside right intracerebral capsular hemorrhage at the age of 37 years. A physical examination revealed the patient’s height as 166.2 cm, weight as 74 kg (body mass index 26.8 kg/m^2^), poor breast development, no beard, slightly low voice tone, no baldness, and female phenotype. The axillary and pubic hairs were poorly developed. Family history showed hypertension in both parents, but not juvenile hypertension. Her parents were non-consanguineous, and none of her siblings had disorders of sex development. She was neither married nor had a partner. Her blood pressure at the onset of the intracerebral hemorrhage was unknown, but her hypokalemia had been approximately 3.0 mmol/L for more than 5 years. Based on the information in her medical questionnaire, menarche occurred at approximately 13 years of age with regular menstrual cycles thereafter, and menopause occurred at the age of 48 years. The patient underwent blood tests for an analysis of the adrenal, gonadal, and pituitary hormones as well as the biomarkers of the renin-angiotensin-aldosterone system (Table 1).

**Table 1:** Results of biochemical and endocrine tests

Laboratory examinations	Results	Reference range (Male Reference range)
Na^+^ (mmol/L)	147	135–147
K^+^ (mmol/L)	3.0	3.5–5.0
Cl^-^ (mmol/L)	107	98–108
Pregnenolone (nmol/L)	30.7	(0.6–4.7)
Progesterone (nmol/L)	21.6	(0.0–1.9)
Deoxycorticosterone (pmol/L)	5858	91–997
Corticosterone (nmol/L)	308.0	0.3–24.5
17-hydroxypregnenolone (nmol/L)	3.9	1.4–35.6
11-deoxycortisol (nmol/L)	3.8	0.3–1.7
DHEA-S (nmol/L)	173	(416–4611)
Androstenedione (nmol/L)	0.2	(0.9–4.2)
Testosterone (nmol/L)	< 0.1	(0.4–1.6)
Estradiol (pmol/L)	91.9	(36.8–147.1)
ACTH (pmol/L)	37.1	1.6–14.1
Cortisol (nmol/L)	80	102–535
PAC (nmol/L)	0.89	0.01–0.67
PRA (ng/L/s)	0.1	0.1–0.8
LH (IU/L)	18.42	(0.79–5.72)
FSH (IU/L)	65.63	(2.00–8.30)
Prolactin (µg/L)	8.99	(3.58–12.78)
24-h urinary cortisol (nmol/24-h)	75	160–1111
24-h urinary metanephrine (nmol/24-h)	304	254–1420
24-h urinary normetanephrine (nmol/24-h)	710	546–1528
24-h urinary potassium (mmol/24-h)	41.6	25.0–50.0

*DHEA-S* Dehydroepiandrosterone sulfate, *ACTH* Adrenocorticotropic hormone, *PAC* Plasma aldosterone concentration, *PRA* Plasma renin activity, *LH* Luteinizing hormone, *FSH* Follicle stimulating hormone.

**Fig. 1:**
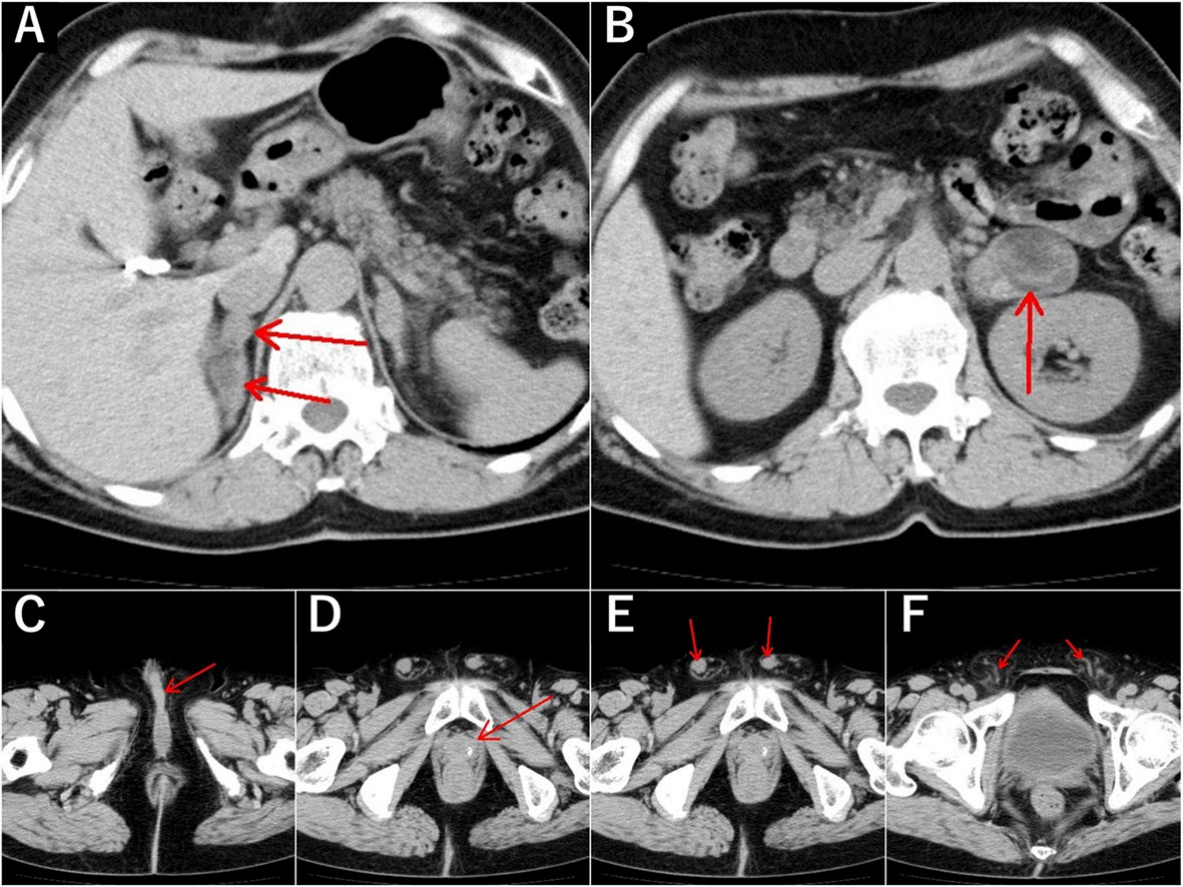
Abdominal and pelvic computed tomography scan. **A**, **B** Both adrenal glands are enlarged. **C** The urethral spongiosa is incompletely developed. **D** Prostate-like structures with calcification are present. **E** Testicular development is incomplete. **F** Testicular veins are present

Blood tests showed high levels of adrenocorticotropic hormone (ACTH), follicle-stimulating hormone (FSH), and luteinizing hormone (LH), low levels of cortisol, dehydroepiandrosterone sulfate, and testosterone, high ARR, and hypokalemia. Computed tomography of the abdomen and pelvis showed bilateral adrenal gland enlargement (the adrenal glands were 46.5 ⋅ 33.2 ⋅ 30.5 mm on the left and 19.9 ⋅ 43.1 ⋅ 23.2 mm on the right), immature urethral spongiosa, prostate-like structures, immature intra-abdominal testes, and testicular veins, but no ovaries or uterus (Fig. 1). The high FSH and LH were thought to be due to gonadal insufficiency.

Subsequently, as 17OHD was strongly suspected, further evaluation was recommended. Thereafter, a short synacthen test, karyotyping, and *CYP17A1* gene analysis were performed. In the short synacthen test, the intravenous administration of synthetic ACTH (250 µg) did not markedly increase cortisol (baseline 80 nmol/L; after 60 min, 91 nmol/L) and 17-hydroxyprogesterone (baseline 4.0 nmol/L; after 60 min, 4.8 nmol/L) levels compared to the baseline levels, suggesting that the pathway catalyzed by 17α-hydroxylase was impaired.

The patient’s karyotype was 46, XY. Genetic analysis revealed heterozygous mutations in the *CYP17A1* gene as follows: (1) c.157_159delTCC, p.Phe54del and (2) c.1118 A > T, p.His373Leu. Therefore, the patient was diagnosed with 17OHD. We informed the patient of the diagnosis with ethical considerations. After the diagnosis, the patient stopped taking antihypertensive medication and was started on oral hydrocortisone (15 mg/day), which is the cornerstone of CAH treatment. Hydrocortisone was taken internally, 10 mg in the morning and 5 mg in the evening. After the appropriate treatment, the blood pressure and serum potassium normalized without antihypertensive therapy.

## Discussion and conclusions

In the present case, elevated aldosterone levels and ARR on a previous laboratory test suggested the diagnosis of PA. However, the patient had findings suggestive of low androgen and inadequate glucocorticoid secretion, which were incompatible with the PA diagnosis. The patient was eventually diagnosed with 17OHD via endocrine profiling, short synacthen test, karyotyping, and genetic analysis. Genetic analysis is important for the definitive diagnosis of 17OHD [7]. In our patient, compound heterozygous mutations (c.157_159delTCC, p.Phe54del and c.1118 A > T, p.His373Leu) were detected in the *CYP17A1* gene, which are frequently reported in Asian countries such as Korea, Japan, and China [8]. Since her karyotype was 46, XY, and she did not have ovaries or a uterus, we realized she had lied in her medical interview.

In 1966, Biglieri et al. reported the first case of 17OHD in a 35-year-old patient [9]. Mutations in the *CYP17A1* gene result in loss of 17α-hydroxypregnenolone/17,20 lyase, causing a decrease in 17α-hydroxyprogesterone and dehydroepiandrosterone, with a concomitant decrease in the production of steroids such as androstenedione, testosterone, 11-deoxycortisol, and cortisol. The accumulation of the substrates pregnenolone and progesterone promotes the 21- and 11β-hydroxylation steps of the mineralocorticoid pathway, resulting in increased levels of 11-deoxycorticosterone, corticosterone, and 18-hydroxycorticosterone. The mineralocorticoid effects of 11-deoxycorticosterone are potent, causing sodium and water retention, hypokalemia, alkalosis, and hypertension. As a result, the renin-angiotensin system is usually suppressed and aldosterone synthesis is reduced, resulting in hyporeninemic hypoaldosteronism [5]. However, our patient showed normal aldosterone levels with suppressed plasma renin activity, similar to contradictory results with normal or high aldosterone levels despite the suppression of plasma renin activity from previous reports [10]. In two previous reports of p.His373Leu heterozygous mutations, renin levels (reference 1.32–3.95 ng/mL/h) were 0.53 ng/mL/h and 0.10 ng/mL/h, whereas aldosterone levels (reference 10–160 pg/mL) were 209 pg/mL and 140 pg/mL, respectively [10]. In contrast, the patient with p.His373Leu homozygous mutation was unable to convert deoxycorticosterone to corticosterone or aldosterone, had low 11β-hydroxylase and aldosterone synthase activity, and reported very low aldosterone levels [11]. In addition, the p.His373Leu mutation has been reported to abolish 17α-hydroxylase activity in an in vitro study [12]. Therefore, it is possible that the patient with p.His373Leu heterozygous mutation may not have sufficiently suppressed aldosterone synthase activity compared to patients with homozygous mutations.

In the present case, only aldosterone was measured by immunoassay, whereas progesterone, deoxycorticosterone, and corticosterone were measured by mass spectrometry. Therefore, we tested whether aldosterone measured with an aldosterone assay kit (SPAC®-S Aldosterone kit) had any effect on cross-reactivity with other steroid hormones. The results showed that the sum of all other steroid hormones measured as aldosterone was significantly different from the measured aldosterone. In other words, the aldosterone value (0.358037 mmol/L) still remained normal after subtracting the false positive value (0.097063 nmol/L) from the actual value (0.4551 nmol/L) (Table 2). The other mineralocorticoids were frankly elevated (mainly corticosterone), and as a result, renin activity was suppressed. The patient was, in fact, producing aldosterone.

**Table 2:** Aldosterone concentration with inferred effects of cross-reactions

Laboratory examinations	Baseline	SPAC®-S Aldosterone kit	
		Crossover rates (%)	Concentrations that could have been measured as aldosterone (nmol/L)
Progesterone (nmol/L)	21.6	0.008	0.001728
Deoxycorticosterone (pmol/L)	5870	0.05	0.002935
Corticosterone (nmol/L)	308	0.03	0.0924
The total concentration that could have been measured as aldosterone (nmol/L)			0.097063
Large discrepancy between the theoretical and measured values			⇕
Measured aldosterone concentration (nmol/L)			0.4551

Because our patient had hypokalemia and a high ARR, we initially considered the case as that of typical PA. Moreover, because of the patient’s advanced age, CAH was not a serious consideration. However, the patient was ultimately diagnosed with 17OHD due to 46, XY karyotype, testicular disorders of sex development, hypokalemia, hyperkaliuresis, low renin activity (suggesting hypermineralocorticism), adrenocortical insufficiency (based on biological data alone), and bilateral adrenal enlargement. In conclusion, the disease 17OHD may resemble PA and presents with high ARR. The combination of a high ARR and low cortisol level should trigger the consideration of 17OHD.

## Data Availability

The datasets used and analyzed during the current study are available from the corresponding author on reasonable request.

## Abbreviations

17OHD: 17α-hydroxylase/17,20-lyase deficiency
ACTH: Adrenocorticotropic hormone
ARR: Aldosterone/renin ratio
CAH: Congenital adrenal hyperplasia
*CYP17A1*: Cytochrome P450 family 17 subfamily A member 1
FSH: Follicle stimulating hormone
LH: Luteinizing hormone
PA: Primary aldosteronism
